# Levofloxacin versus clarithromycin for *Helicobacter pylori* eradication: are 14 day regimens better than 10 day regimens?

**DOI:** 10.1186/s13099-022-00502-3

**Published:** 2022-06-06

**Authors:** Eman T. Azab, Abrar K. Thabit, Sean McKee, Aymen Al-Qiraiqiri

**Affiliations:** 1 King Fahd Armed Forces Hospital, Jeddah, Saudi Arabia; 2 Pharmacy Practice Department, Faculty of Pharmacy, King Abdulaziz University, Jeddah, Saudi Arabia; 3 Pharmaceutical Care Department, King Fahd Armed Forces Hospital, Jeddah, Saudi Arabia

**Keywords:** Levofloxacin, Clarithromycin, *Helicobacter pylori*, Peptic ulcer

## Abstract

**Background:**

*Helicobacter pylori* eradication by the conventional clarithromycin therapy has largely dropped in the recent years possibly due to antimicrobial resistance. Hence, levofloxacin-based regimen has been used as salvage therapy. However, data regarding its effectiveness on eradication are controversial. This study aimed to compare the eradication rate of levofloxacin-based regiment to that of the conventional first-line clarithromycin regimen.

**Methods:**

Patients diagnosed with *H. pylori* infection and treated with levofloxacin triple therapy or clarithromycin-based regimen for 10 or 14 days were included. Patients were excluded if they used antibiotics or proton pump inhibitors within 4 or 2 weeks, respectively, of the *H. pylori* eradication confirmation test. *H. pylori* eradication rate was assessed, as well as the impact of diabetes and esophagogastroduodenoscopy (EGD) findings.

**Results:**

Of 245 patients, 145 were in the levofloxacin group and 100 in the clarithromycin group. Most patients in either group received therapy for 14 days vs. 10 days (*P* = 0.002). Levofloxacin-based treatment was associated with a higher eradication rate compared with clarithromycin-based treatment (74.5 vs. 62%, respectively; *P* = 0.04). The 14 day levofloxacin-based regimen resulted in the highest eradication rate, followed by the 14 day regimen of clarithromycin (80.9 vs. 66.3%; *P* = 0.03). The 10 day regimens exhibited 62.7 and 41.2% eradication rates, respectively (*P* = 0.12). *H. pylori* eradication was not affected by diabetes or EGD findings (*P* = 0.98 and 0.3, respectively).

**Conclusions:**

Results from this study support the use of a levofloxacin-based regimen as a first-line therapy in the treatment of *H. pylori* infection for 14 days regardless of diabetes and EGD findings.

## Introduction

*Helicobacter pylori* infection is one of the most common gastrointestinal infections. It is considered the primary cause of gastritis, peptic ulcer, gastric cancer, and mucosa-associated lymphoma [1]. Infection with this Gram-negative microaerophile has been handled using a mixture of antibiotics and proton pump inhibitors (PPIs) [2]. The conventional first-line treatment is triple therapy, which includes a PPI, clarithromycin, and amoxicillin [3].

Globally, the rate of eradication with this conventional therapy has dropped to less than 80% presumably as a result of increased resistance to clarithromycin [4]. Saudi Arabia witnesses increasing emergence of antibiotic resistance to classical treatment plans, and consequently, it is endorsed that the common use of metronidazole and clarithromycin in medical practice must be limited and switched to alternative regimens [2, 5]. The latest available data from the literature in 2008 showed a resistance to metronidazole of up to 69%, while resistance to clarithromycin and amoxicillin were at 21% and 0%, respectively [6]. It is assumed that these rates may have risen with the continuous use of these antibiotics in *H. pylori* infections.

The main determinants of successful *H. pylori* eradication include assessment of pretreatment resistance to antimicrobial agents, treatment duration, regimen type, and patient compliance [7]. Additionally, there has been a debate about the relationship between *H. pylori* infection and diabetes as some studies showed an increased risk of infection in diabetic patients and a lower eradication rate [8, 9].

In patients who fail the first-line regimen, clarithromycin can be substituted with levofloxacin. [10] Levofloxacin is a broad spectrum flouroquinolone that covers Gram-positive and Gram-negative bacteria, as well as atypical bacteria. [11] Levofloxacin-based triple therapy for *H. pylori* infections has been proven to be functional as a second or third-line rescue regimens with a reported eradication rate of 75 to 90% [4, 12]. Nevertheless, the emergence of resistance to levofloxacin was associated with a diminished *H. pylori* eradication rate [13]. Notably, resistance testing is not routinely performed to guide prescribing in the treatment of *H. pylori* infection [14]. While levofloxacin-based triple therapy is widely used, there are inconsistencies within the published data regarding its effectiveness in eradicating *H. pylori* [15]. Therefore, the present study was designed to assess the eradication rate of levofloxacin as salvage therapy and compare it with that of the conventional first-line regimen comprised of clarithromycin, as well as evaluate the impact of diabetes and esophagogastroduodenoscopy (EGD) findings on such outcome.

## Methods

### Study design and patients

 This was a retrospective cohort study conducted in a tertiary care hospital, (King Fahd Armed Forces Hospital) in Jeddah, Saudi Arabia. The study protocol was approved by the institutional review board of the hospital. The data were collected from patients’ electronic medical records.

Adult patients aged 18 years or older who were diagnosed with *H. pylori* infection by positive urea fecal antigen test, rapid urease test (*Campylobacter*-like organism test; CLO test), or histological test between January 1, 2017 to December 31, 2018 were included. Patients were excluded if they used antibiotics or PPIs within 4 weeks or 2 weeks, respectively, of the *H. pylori* eradication confirmation test. Patients who did not take the full course of therapy or who did not have follow-up test with urea fecal antigen test (CTK Onsite, San Diego, CA, USA), rapid urease test (CLO test) or histological result for *H. pylori* eradication were excluded.

Included patients were treated with either levofloxacin triple therapy (levofloxacin 500 mg once daily plus amoxicillin 1 g every 12 h or metronidazole 500 mg every 8 h plus PPI) or with clarithromycin-based therapy (clarithromycin 500 mg every 12 h plus amoxicillin 1 g every 12 h or metronidazole 500 mg every 8 h plus PPI) for 10–14 days. The PPI used in our patient population was either esomeprazole or pantoprazole.

### Study endpoints

The primary end point was the eradication rate of *H. pylori* at the end of either a 10 day or 14-day treatment course. This was defined as a negative result in the follow up *H. pylori* test. Secondary endpoints were the influence of diabetes, EGD findings, and secondary drugs (e.g., amoxicillin and type of PPI) on *H. pylori* eradication.

### Statistical analysis

Sample size was calculated using a two-tailed alpha test with a significance level of 0.05 and 90% power to detect 20% difference in the eradication rate of the two regimens. The total number of patients required to prove the hypothesis was 245. Patients ages were expressed as mean ± standard deviation (SD) and compared using *t*-test as Shapiro-Wilk test for normality showed normal distribution. On the other hand, categorical variables were expressed as numbers and percentages and compared using Chi-square test. A *P* value of < 0.05 was considered statistically significant. SPSS version 24.0 (SPSS, Inc., Chicago, Illinois, USA) was used for statistical analysis.

## Results

A total of 245 patients were included in the study, 145 in the levofloxacin group and 100 in the clarithromycin group. No difference in the baseline characteristics of the two groups was observed, except for the duration of therapy, where most patients in both groups received therapy for 14 days rather than 10 days (*P* = 0.002) (Table 1). Twenty-eight patients (19.3%) in the levofloxacin group vs. 24 patients (24%) in the clarithromycin group had diabetes. The most common EGD abnormalities were found in the stomach in both groups, which were in the form of gastric erosion, gastric ulcer, gastritis, or nodular gastritis. Many patients also had abnormalities in more than one site, such as gastritis with either esophagitis or duodenitis. One patient in the levofloxacin group had adenocarcinoma.

**Table 1:** Baseline characteristics of patients

Characteristic	Levofloxacin, n = 145	Clarithromycin, n = 100	*P* value
Age	44.8 ± 12.9	45.7 ± 13.9	0.6
Sex (female)	89 (61.4)	60 (60)	0.8
Diagnostic test			
• CLO test • Histopathology • Stool antigen	51 (35.2) 71 (49) 23 (15.9)	42 (42) 48 (48) 10 (10)	0.3
Follow up test			
• CLO test • Histopathology • Stool antigen	10 (6.9) 14 (9.7) 121 (83.4)	7 (7) 16 (16) 77 (77)	0.3
Diabetes	28 (19.3)	24 (24)	0.4
EGD findings			
• Esophagus • Stomach • Duodenum • Multisite • None	2 (1.4) 65 (44.8) 7 (4.8) 22 (15.2) 49 (33.8)	1 (1) 54 (54) 3 (3) 20 (20) 22 (22)	0.3
Duration of the therapy			
• 10 days • 14 days	51 (35.2) 94 (64.8)	17 (18) 83 (83)	0.002

Data are presented as mean ± SD or n (%)

*CLO* *Campylobacter*-like organism test (rapid urease test), *EGD* esophagogastroduodenoscopy

**Fig. 1:**
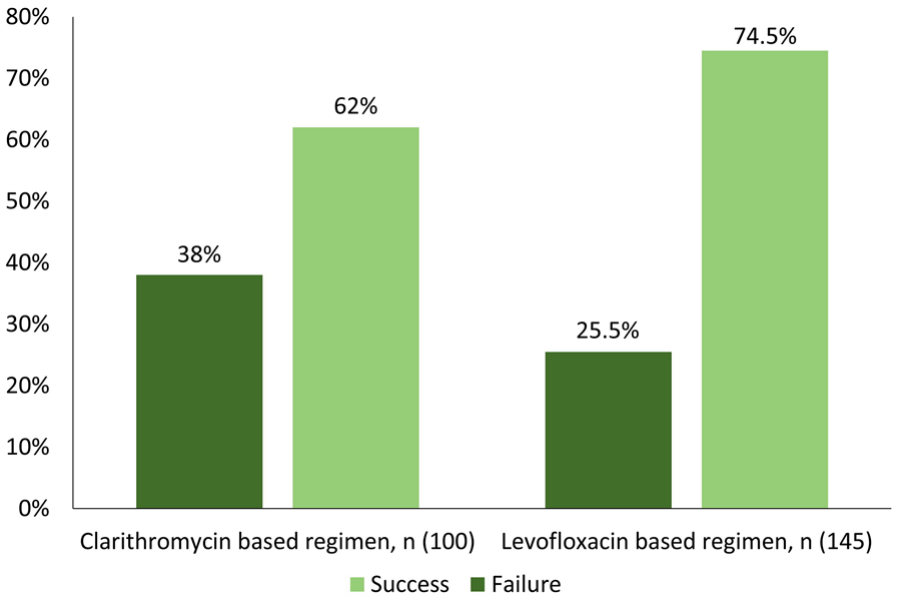
Overall *H. pylori* eradication rates by levofloxacin and clarithromycin regimens

Levofloxacin-based treatment was associated with a higher eradication rate compared with clarithromycin-based treatment (74.5% vs. 62%, respectively; *P* = 0.04) (Fig. 1). Table 2 shows the comparison between the two regimens given for 10 or 14 days. The highest eradication rate was observed with the 14-day levofloxacin-based regimen at 80.9%, followed by the 14-day regimen of clarithromycin at 66.3% (*P* = 0.03). On the other hand, the 10-day regimens resulted in 62.7% and 41.2% eradication rates for both drugs, respectively (*P* = 0.12). The overall difference between all regimens was significant (*P* = 0.02). This significance persisted when regimens were compared as small groups based on each secondary drug received (Table 3) and when the small proportion of patients who received metronidazole were excluded (Table 4) (*P* = 0.03 in both comparisons).

**Table 2:** Effect of the duration of treatment on *H. pylori* eradication of each regimen

Duration of Therapy	Outcome	Levofloxacin– based regimen (n = 145)	Clarithromycin– based regimen (n = 100)	*P* value
10 days	Success	32 (62.7)	7 (41.2)	0.12
	Failure	19 (37.3)	10 (48.8)	
14 days	Success	76 (80.9)	55 (66.3)	0.03
	Failure	18 (19.1)	28 (33.7)	

Data are presented as n (%)

Overall difference between all regimens was significant (*P* = 0.02)

**Table 3:** Effect of the duration of treatment on *H. pylori* eradication of each regimen and secondary drug

Duration of Therapy	Outcome	LAP (n = 43)	LAE (n = 95)	LMP (n = 2)	LME (n = 5)	CAP (n = 45)	CAE (n = 52)	CME (n = 3)
10 days	Success	18 (78.3)	14 (50)	NA	NA	2 (22.2)	5 (62.5)	NA
	Failure	5 (21.7)	14 (50)	NA	NA	7 (77.8)	3 (37.5)	NA
14 days	Success	14 (70)	58 (86.6)	1 (50)	3 (60)	23 (63.9)	32 (72.7)	0 (0)
	Failure	6 (30)	9 (13.4)	1 (50)	2 (40)	13 (36.1)	12 (27.3)	3 (100)

*LAP* levofloxacin, amoxicillin, and pantoprazole; *LAE* levofloxacin, amoxicillin, and esomeprazole, *LMP* levofloxacin, metronidazole, and pantoprazole, *LME* levofloxacin, metronidazole, and esomeprazole, *CAP* clarithro,mycin, amoxicillin, and pantoprazole, *CAE* clarithromycin, amoxicillin, and esomeprazole, *CME* clarithromycin, metronidazole, and esomeprazole

Data are presented as n (%)

Overall difference between all regimens was significant (*P* = 0.03)

None of the included patients received clarithromycin, metronidazole, and pantoprazole

**Table 4:** Effect of the duration of treatment on *H. pylori* eradication of each regimen and secondary drug excluding metronidazole-containing regimens

Duration of Therapy	Outcome	LAP (n = 43)	LAE (n = 95)	CAP (n = 45)	CAE (n = 52)
10 days	Success	18 (78.3)	14 (50)	2 (22.2)	5 (62.5)
	Failure	5 (21.7)	14 (50)	7 (77.8)	3 (37.5)
14 days	Success	14 (70)	58 (86.6)	23 (63.9)	32 (72.7)
	Failure	6 (30)	9 (13.4)	13 (36.1)	12 (27.3)

*LAP* levofloxacin, amoxicillin, and pantoprazole, *LAE* levofloxacin, amoxicillin, and esomeprazole, *CAP* clarithromycin, amoxicillin, and pantoprazole, *CAE* clarithromycin, amoxicillin, and esomeprazole

Data are presented as n (%)

Overall difference between all regimens was significant (*P* = 0.03)

**Fig. 2:**
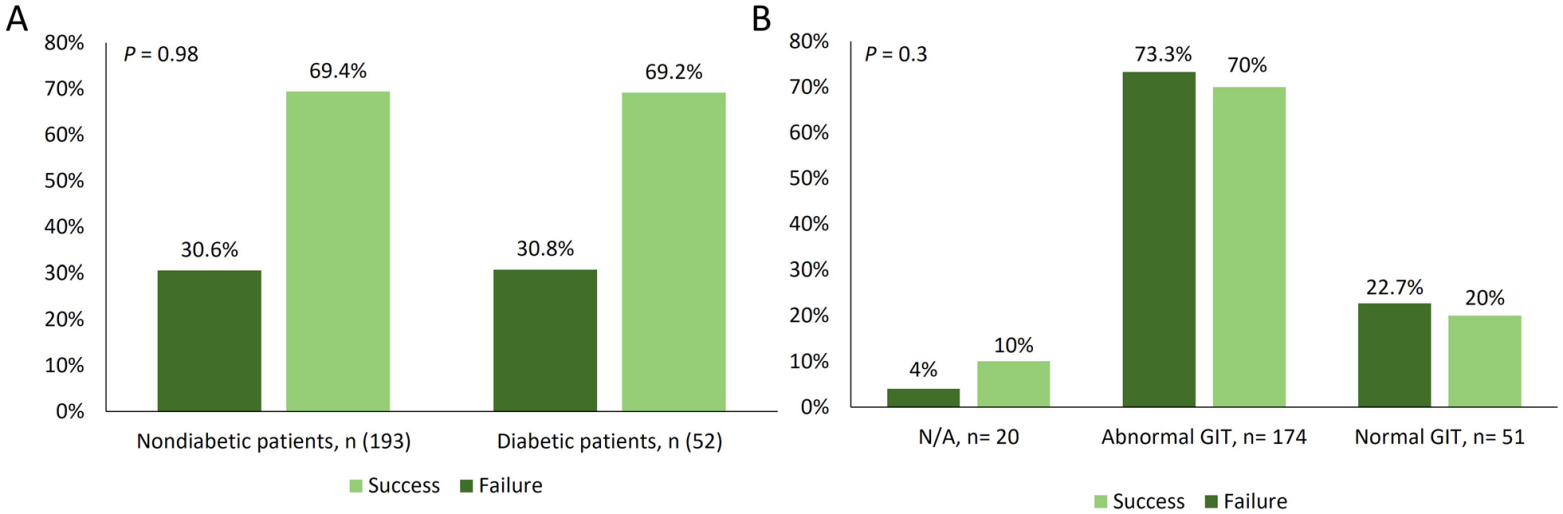
The influence of diabetes and esophago-gastroduodenoscopy (EGD) findings on *H. pylori* eradication (GIT, gastrointestinal tract; N/A, not applicable)

**Fig. 3:**
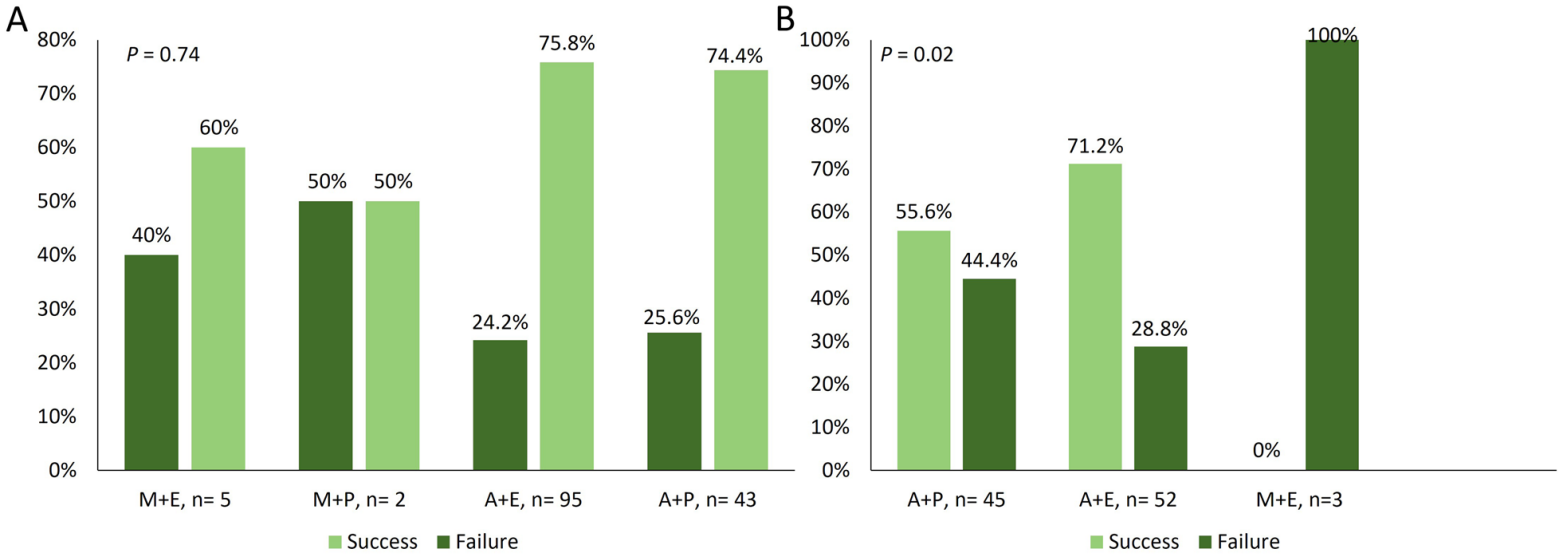
The effects of secondary drugs on *H. pylori* eradication with **A** levofloxacin and **B** clarithromycin. *A* amoxicillin, *E* esomeprazole, *M* metronidazole, *P* pantoprazole

In terms of the impact of diabetes and EGD findings, results showed that successful eradication of *H. pylori* was not affected by either (*P* = 0.98 and 0.3, respectively) as illustrated in Fig. 2. When the effect of different secondary drugs on eradication rate was evaluated, only the combination of amoxicillin plus esomeprazole with clarithromycin was associated with higher eradiation rate (71.2%) compared with the other combinations (*P* = 0.02) (Fig. 3). On the contrary, all secondary drugs were equally effective with levofloxacin (*P* = 0.74).

## Discussion

Since the emergence of falling eradication rates and the need for new medications, the use of levofloxacin has been recommended. Resistance to metronidazole and clarithromycin, which are the cornerstones of traditional triple treatments, makes it difficult to find an effective alternative [14]. One of the suggested salvage regimens in second-line treatment is 10–14 day levofloxacin-based triple therapy. When compared to clarithromycin-based treatment, our study showed that levofloxacin-based treatment was linked with a higher incidence of treatment success (74.5 vs. 62%). Similar results were confirmed by Haji-Aghamohammadi et al. who reported the superiority of levofloxacin triple regimen to clarithromycin-based regimen (75 vs. 51.7% and 80.4 vs. 57.4%) according to intention- to-treat and per protocol analyses, respectively [16]. Another large randomized trial of 300 patients who were treated with either a standard clarithromycin regimen or a levofloxacin triple therapy showed a higher eradication rate of 87% with levofloxacin-containing therapy compared to standard regimens with eradication rates of 72 and 75% (clarithromycin with esomeprazole and either amoxicillin or metronidazole, respectively) [11].

Discrepancies between trial results could be also attributed to ethnic differences, although drug dosage and treatment duration should also be considered. Such impact was suggested by Silva et al. who conducted a study on 66 Brazilian patients who had *H. pylori* infection, did not receive prior treatment, and were treated with levofloxacin triple-based regimen (levofloxacin, amoxicillin, and lansoprazole) showed an eradication rate of 73% (95% CI, 62–84%) compared with 82.7% (95% CI, 79–86%) reported with classic regimen of clarithromycin, amoxicillin, and a PPI [17]. This finding could be explained by an earlier Brazilian study that found higher rates of resistance by *H. pylori* to levofloxacin (23%) than to clarithromycin (8%) [18].

In terms of duration of therapy, the present study found that the eradication rate of *H. pylori* who failed the first-line was 62.7% and 80.9% for 10-day and 14-day levofloxacin-based regimen, respectively. On the other hand, the use of a clarithromycin-based regimen for 10 or 14 days resulted in eradication rates of 41.2% and 66.3%, respectively (*P* = 0.02). These differences suggest that the 14-day levofloxacin-based triple therapy is more effective in eradicating *H. pylori* infection than clarithromycin-based therapy for either 10 or 14 days treatment course. Regarding the optimal duration of levofloxacin triple salvage treatment, Di Caro et al. compared two types of 10-day and two types of 7 day PAL regimens in Italy and found significantly higher efficacy with longer duration (88 vs. 78%) [14]. This finding was confirmed by an RCT from Turkey which reported significantly higher efficacy with longer duration of PAL as first-line treatment (72% with 14 day regimen vs. 34% with 7 day regimen) [19]. Furthermore, according to Gisbert et al. systemic review and meta-analysis, when levofloxacin– amoxicillin–PPI combination was given for 7 and 10 days, the mean eradication rate was 68% (95% CI, 62–75%) and 80% (95% CI, 77–83%) (*P* < 0.001) [15].

Our findings revealed that the presence of diabetes as a comorbid condition had no effect on *H. pylori* eradication. These findings are consistent with those reported by Kato et al., where the eradication failure was reported in 3.7% of diabetic patients and 2.5% of patients without diabetes. Although patients with diabetes were more likely to have eradication failure, the difference was not statistically significant (1.2%; 95% CI, − 0.8–3.2%) [19]. On the other hand, Horikawa et al. showed a significantly higher risk of *H. pylori* eradication failure in patients with diabetes compared with non-diabetic patients (*P* < 0.001) in a meta-analysis of 693 patients, of whom 273 had diabetes) [9]. Therefore, the authors recommended that diabetic patients should be treated for an extended duration or to use a new regimen for *H. pylori* eradication. Several studies explained the potential mechanisms that explain the low rate of effective *H. pylori* eradication among diabetic patients [20, 21] .

Similar to our findings with diabetes, the presence or absence of any GI condition had no impact on the successful eradication of *H. pylori*. Nonetheless, Kalkan et al. demonstrated that treatment failure was higher in the presence of gastric atrophy and intestinal metaplasia [22]. Due to a lack of data on the relationship between the existence and grade of intestinal metaplasia/atrophy and *H. pylori* eradication success, we were unable to explain the specific mechanism behind this outcome.

Based on our results, the eradication rate of *H. pylori* was improved in the presence of amoxicillin plus esomeprazole in the clarithromycin-based regimen patients (*P* = 0.02). In the levofloxacin-based regimens, however, the type of PPI and the inclusion or exclusion of amoxicillin within the regimen had no change on the eradication rate. The use of esomeprazole had a higher cure rate when compared to pantoprazole in a study published by Graham et al. This is a finding they attributed to the greater relative potency of esomeprazole compared to pantoprazole; an observation that was not directly assessed in this present study [23]. However, the finding that esomeprazole-treated patients had high eradication rates in our study (in the clarithromycin-based regimens) is consistent with the findings of Graham et al. and most likely attributable to the high potency of esomeprazole compared to pantoprazole.

The findings of this study must be seen in light of some limitations. Patient compliance was not assessed, though there is no reason to assume a difference in compliance between these two regimens. This is a single center study with low rates of levofloxacin resistance in the region, possibly limiting the generalizability of the results. The choice of PPI was not compared based on the relative potency; possibly skewing the results as some studies state esomeprazole has greater potency than pantoprazole requiring a dose-adjustment for comparison based on equal-potency dosages. Our study favors real-life practice with physicians prescribing typical dosages of each PPI. Lastly, the levofloxacin-based regimen was used as salvage therapy, whereas the clarithromycin group was more likely to be treatment-naïve.

## Conclusions

The highest eradication rate in patients diagnosed with *H. pylori* infection occurred in the 14-day levofloxacin-based regimen. Nonetheless, clinicians should observe potential serious adverse effects associated with fluroquinolone therapy, such as tendinopathy and cardiovascular side effects.

Given the limited scope of the present study, the results should be hypothesis generating. Additional studies should be conducted to further assess the effectiveness of the levofloxacin-based regimen in the eradication of *H. pylori* infections compared with the current first-line clarithromycin-based regimen. The most favorable of the clarithromycin-based regimens was the 14-day clarithromycin regimen that included esomeprazole and amoxicillin. Future studies randomizing and comparing the 14-day course of levofloxacin-based regimen with a 14-day course of clarithromycin-based regimen while accounting for secondary medications are warranted.

### Significance statement

This study compared two different antibiotic regimens for the treatment of peptic ulcer (an infection in the stomach or upper small bowel caused by bacteria). The regimen that involved levofloxacin was more effective than the one that involved clarithromycin (has always been considered as the first line). Additionally, the presence of diabetes or gastrointestinal tract abnormalities did not affect the outcomes of the treatments.

## Data Availability

Data are available upon request from the authors.
